# TCDD Promotes Lung Tumors via Attenuation of Apoptosis through Activation of the Akt and ERK1/2 Signaling Pathways

**DOI:** 10.1371/journal.pone.0099586

**Published:** 2014-06-13

**Authors:** Rong-Jane Chen, Shih-He Siao, Chung-Huei Hsu, Chu-Yung Chang, Louis W. Chang, Chih-Hsiung Wu, Pinpin Lin, Ying-Jan Wang

**Affiliations:** 1 Department of Environmental and Occupational Health, National Cheng Kung University Medical College, Tainan, Taiwan; 2 Graduate Institute of Clinical Medicine, Taipei Medical University, Taipei, Taiwan; 3 Department of Nuclear Medicine, Taipei Medical University Hospital, Taipei, Taiwan; 4 National Environmental Health Research Center, National Health Research Institutes, Zhunan Town, Taiwan; 5 Department of Surgery, School of Medicine, Taipei Medical University-Shuang Ho Hospital, Taipei, Taiwan; 6 Center of Excellence for Cancer Research, Taipei Medical University, Taipei, Taiwan; 7 Division of Environmental Health and Occupational Medicine, National Health Research Institutes, Zhunan Town, Taiwan; 8 Department of Biomedical Informatics, Asia University, Taichung, Taiwan; Henry Ford Health System, United States of America

## Abstract

2,3,7,8-Tetrachlorodibenzo-p-dioxin (TCDD) is a multiple-site, multiple-species carcinogen that induces cancer in multiple organs. The molecular mechanisms underlying TCDD-induced lung tumorigenesis remain unclear. In the present study, a two-stage lung tumorigenesis model was established by administrating a single low dose of 4-(methylnitrosamino)-1-(3-pyridyl)-1-butanone (NNK) combined with TCDD to female A/J mice. The results indicated that TCDD combined with low-dose NNK has a significant tumor-promoting effect compared with TCDD or low-dose NNK alone. Resistance to apoptosis is a hallmark of cancer and is thought to be one of the tumor-promoting mechanisms regulated by TCDD. We performed an additional series of experiments in the normal human bronchial epithelial cell line Beas2B cells, in which TCDD was combined with the apoptosis inducer staurosporine. Our *in vitro* results confirmed that TCDD could rescue cells from apoptosis induced by staurosporine. The inhibition of apoptosis is likely mediated by the activation of the Akt and ERK1/2 pathways, as determined by the effectiveness of pathway-specific inhibitors in abrogating the anti-apoptotic activity of TCDD. In conclusion, we demonstrated that TCDD promoted NNK-induced lung tumorigenesis and revealed that TCDD inhibits staurosporine-induced apoptosis, at least in part, through the Akt and ERK1/2 signaling pathways.

## Introduction

2,3,7,8-Tetrachlorodibenzo-*p*-dioxin (TCDD, dioxin) is the most potent congener in a family of polychlorinated dibenzo-*p*-dioxins and dibenzofurans (PCDDs/PCDFs). These compounds are formed as industrial by-products [1] and are found as persistent contaminants in the environment, food, human tissues, and human milk [2]. Human exposure to dioxins occurs mainly through contaminated food such as cow's milk, milk products, bovine adipose tissue, hen's eggs and fish [3]. Much of the concern regarding TCDD relates to its environmental and biological persistence, which may result in bio-concentration and bio-accumulation as it is transported up the food chain, thereby inducing various toxic effects in humans [4], [5]. The toxic effects induced by TCDD include reproductive and developmental defects, immunotoxicity, liver damage, wasting syndrome, and cancer [6]. The toxicity of TCDD requires the activation of the aromatic hydrocarbon receptor (AhR), which results in transcriptional activation or repression of a diverse array of genes [7]. Of these various toxicities, the carcinogenic effects of TCDD attracted particular concern. Based on the evidence of carcinogenicity in experimental animals and the mechanistic studies *in vitro*, TCDD was classified as a class I human carcinogen by the International Agency for Research on Cancer (IARC) [8]. However, Cole et al. indicated that the increased human cancer risk was only modest for people exposed to TCDD. They indicated that cancer risk increased when TCDD exposure was combined with other environmental factors, such as cigarette smoke [9]. In addition, it has been reported that cigarette smoke has extremely high levels of dioxins, dioxin-like compounds that are agonists of AhR [10]. Therefore, co-exposure of TCDD and other chemicals such as cigarette carcinogens may have synergistic effects in adverse health problems such as cancer.

Considerable evidence has indicated that TCDD is a potent tumor promoter in livers and in the skin of mice [11], [12]. Several studies have also shown that the lung is a target organ for TCDD toxicity. Exposure to high levels of TCDD is associated with the development of chronic obstructive pulmonary disease and lung cancer in humans [13]. In addition, TCDD could increase human lung cancer risk by promoting tumors initiated by exposure to other specific chemicals. For example, male Swiss mice given a single dose of dimethyl-N-nitrosamine (DMN) followed by TCDD for 20 weeks showed an increase in the development of lung alveolar-cell adenomas and carcinomas [14]. Our previous study indicated that TCDD exposure combined with the cigarette smoke carcinogen 4-(methylnitrosamino)-1-(3-pyridyl)-butanol (NNK) significantly increased lung tumor formation in A/J mice [15]. These studies indicated the synergistic effects of TCDD in carcinogenesis; however, the underlying mechanisms remain unclear and require further investigation.

TCDD is not a mutagen and does not interact with DNA. It is not considered directly genotoxic, on the contrary, TCDD acts as a classical tumor promoter [2]. The underlying mechanisms of TCDD in tumor promotion remain unclear; it was proposed that an epigenetic mechanism plays an important role in TCDD-induced tumorigenesis. Previous reports have indicated that TCDD protects MCF-10A cells from EGF-withdrawal-induced apoptosis via the activation of the anti-apoptotic kinase Akt (protein kinase B) and the extracellular signal regulated kinase (ERK) [16], [17]. TCDD causes the autocrine production of TGF-α in an AhR -dependent manner and thereby mimics the action of EGF, implying that TCDD could serve as an anti-apoptosis agent [18]. Furthermore, high concentrations of TCDD (1–25 nM) have been reported to inhibit apoptosis triggered by chemicals or UV irradiation in hepatocytes [19]. The anti-apoptotic effect of TCDD in hepatocytes involves the repression of p53 functions through varies mechanisms [20]–[22]. Another study showed that staurosporine (STS)-induced apoptosis could be attenuated by TCDD in the mouse myoblastoma cell line C2C12, indicating that TCDD could activate a calcineurin A-sensitive pathway and led to NFκB activation followed by the induction of mitochondrial stress signaling [23]. Thus, the inhibition of apoptosis and increased cell proliferation triggered by TCDD may play central roles in its ability to promote tumors [24]. Lung cancer is the most commonly diagnosed cancer and is the leading cause of cancer death in males and the second-leading cause of cancer death in females. One factor in its etiology is exposure to environmental toxins, such as cigarette smoke and TCDD [25]. As previously mentioned, several signaling pathways have been proposed to mediate TCDD-related tumor-promoting effects in cancer cells [16], [17], [21], [22], [26]. However, only a limited number of studies have been conducted in animal and cultured cells models to investigate the lung tumor-promoting and anti-apoptotic effects of TCDD. Understanding the mechanism of lung tumor-promotion by TCDD could be helpful in designing strategies for lung cancer prevention and therapy for people who are continuously exposed to TCDD. In our current study, we used the NNK-initiated lung tumorigenesis model to study the lung tumor-promoting effect of TCDD in female A/J mice and investigated the anti-apoptotic mechanism of TCDD using the normal human bronchial epithelial cell line, Beas2B cells. Our results demonstrated that TCDD promotes NNK-induced lung tumorigenesis and revealed that TCDD inhibits STS-induced apoptosis through the Akt and ERK1/2 signaling pathways.

## Materials and Methods

### Chemicals and reagents

TCDD and NNK were obtained from Toronto Research Chemicals Inc. (TRC, North York, Ontario, Canada). TCDD was dissolved in dimethylsulfoxide (DMSO) and stored in the dark at −20°C until use. NNK was dissolved in normal saline solution immediately before use. The ERK1/2 inhibitor U0126 and the PI3K/AKT inhibitor LY294002 were obtained from Cell Signaling (Beverly, MA, USA). Primary antibodies against Akt, phospho-Akt, PI3K, phospho-PI3K, phospho-PDK1, phospho-PTEN (phosphatase and tensin homolog), caspase-8, cleaved caspase-8, PARP, ERK1/2, phospho-ERK1/2, and GAPDH, as well as horseradish peroxidase (HRP)-conjugated anti-mouse and anti-rabbit secondary antibodies, were purchased from Cell Signaling (Beverly, MA, USA). Bad, Bcl-xl, and Bcl-2 antibodies were purchased from R&D Systems (Minneapolis, MN, USA). Pro-caspase-3 and cleaved caspase-3 antibodies were purchased from Epitomics, Inc. (Burlingame, CA, USA).

### Animals, treatments, and experimental design

**Figure 1:**
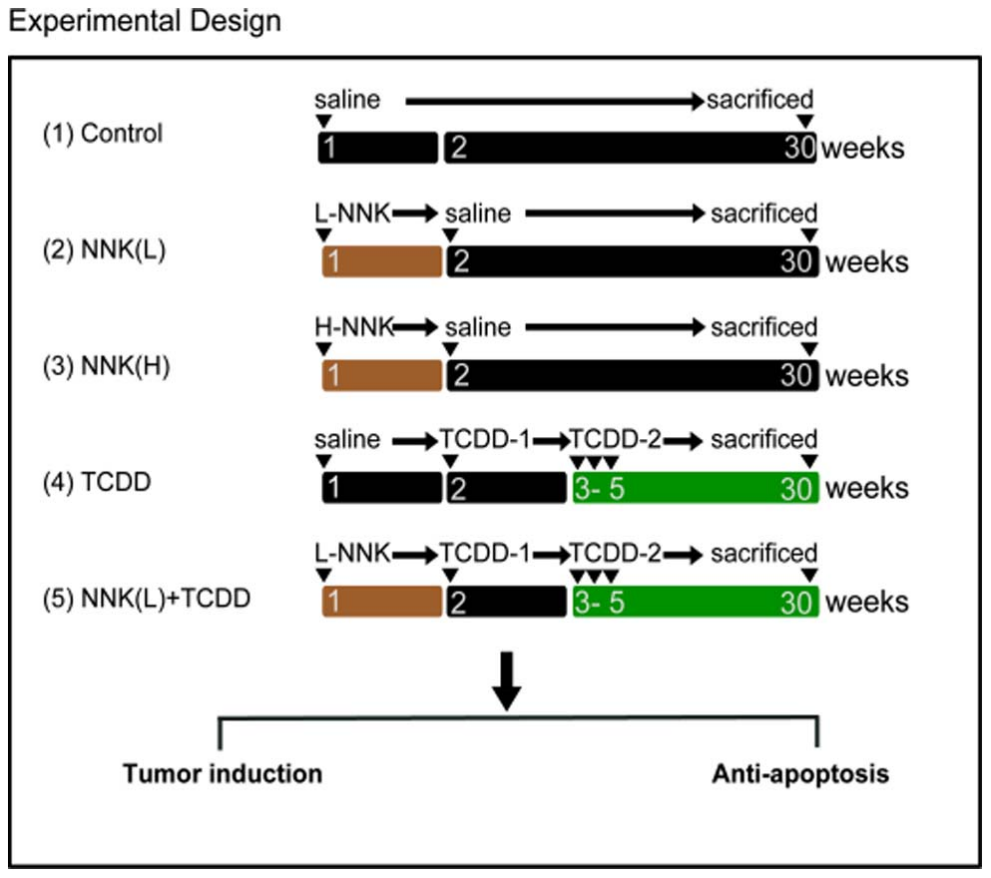
Experimental design. Female A/J mice were divided into 5 groups. Group 1 is the control group, in which mice were given normal saline once a week for 4 weeks. Group 2 is the low-dose NNK group (NNKL), in which mice were treated with NNK (1 mg/0.1 ml/mouse, i.p.) in week 1 of the experiment. Group 3 received a single injection of high-dose NNK (2 mg/0.1 ml/mouse, NNK(H)). Group 4 received a loading dose of TCDD (5 µg kg b.w., i.p.) in week 2 and a maintenance dose of TCDD (1.42 µg kg b.w., i.p) 3 times a week starting in week 3 for 3 weeks. Group 5 received a single injection of low-dose NNK [NNK(L)] in week 1 of the experiment, followed by a loading dose of TCDD and a maintenance dose of TCDD similar to that of group 4. The experiment was terminated at week 30 to evaluate lung tumor formation.

Six-week-old female A/J mice, which were acquired from the Laboratory Animal Center of the National Cheng Kung University Medical College, were housed (five mice per cage) in a pathogen-free environment, maintained on Lab Diet 5010 chow (PMI Feed, Inc, USA) at 24±2°C and 50±10% relative humidity, and subjected to a 12 h light/12 h dark cycle. The animal studies were approved by the Laboratory Animal Center of the National Cheng Kung University Medical College and performed according to the local guidelines for animal care and protection. The experimental design for the treatment groups and for the duration of exposure is shown in Figure 1.

At 7 weeks of age, the mice were randomly divided into 5 groups. The treatments of the 5 groups were as follows: (1) control group: i.p. normal saline, 0.1 ml, n = 5; (2) NNK low dose group [NNK(L)]: a single i.p. injection of 1 mg NNK, n = 10; (3) NNK high dose group [NNK(H)]: a single injection of 2 mg NNK, n = 10; (4) TCDD group: the mice were given a loading dose of TCDD in week 2, followed by a weekly maintenance dose of TCDD for 3 weeks, n = 10; (5) NNK low dose combined with TCDD group: the mice were given a single low dose of NNK(L) (1 mg/mouse) in week 1, followed by a loading dose of TCDD, and were then given weekly maintenance doses of TCDD for 3 weeks, n = 10.

NNK was given intraperitoneally in week 1 of the experiment (Groups 2, 3, and 5). In week 2, the loading dose of TCDD (5 µg/kg b.w., i.p.) was given, and the maintenance dose of TCDD (1.42 µg/kg b.w., i.p.) was then given 3 times a week for an additional 3 weeks (Groups 4 and 5, weeks 3–5). The doses of TCDD were based on those described in our previous study, which showed successful enhancement of lung tumorigenesis in mice. At the end of the experiment (30 weeks), all of the mice were euthanized under ether anesthesia. The lung tissues were dissected, weighed, and examined for the presence of gross tumors and were then either sectioned and fixed in 10% paraformaldehyde or frozen in liquid nitrogen.

### Histopathological analysis and immunohistochemistry (IHC)

The whole lungs from 3 mice of each group were kept at −80°C for western blotting analysis. The lungs from the remaining mice of each group were preserved in 10% buffered formalin. Lung tissue sections (4 µm) were sliced from paraffin-embedded, formalin-fixed lungs and stained with hematoxylin and eosin (H&E). The lung tumor measurements and immunohistochemistry for PCNA staining were performed according to the methods described in our previous study [27].

### Beas2B cell culture conditions

Immortalized human bronchial epithelial cell line, Beas2B cells, were purchased from ATCC (American Type Culture Collection, Manassas, VA, USA) and were maintained in DMEM medium (Life Technologies, Inc., Gaithersburg, MD, USA) supplemented with 100 U/ml penicillin, 100 µg/ml streptomycin (Life Technologies, Inc., Gaithersburg, MD, USA), and 5% heat-inactivated fetal calf serum (HyClone, South Logan, UT, USA). The Beas2B cells were incubated at 37°C in a 5% CO_2_ atmosphere incubator.

### Assessment of apoptosis

One of the early characteristics of apoptosis is the rapid translocation and accumulation of the membrane phospholipid phosphotidylserine (PS) from the cytoplasmic interface to the extracellular surface. To detect this apoptotic marker, cells were trypsinized, washed with 1XPBS, centrifuged, and resuspended in 1X Annexin V binding buffer (10 mM HEPES, pH 7.4, 0.14 M NaCl, and 2.5 mM CaCl_2_) containing 5 µl of Annexin V-FITC (Becton Dickinson, San Jose, CA) at room temperature for 15 min. An additional 400 µl of 1X binding buffer was added to stop the reaction, and the percentage of Annexin V-positive cells was measured by a FACScan cytometer (Becton Dickinson, San Jose, CA).

### Western blot analyses

The total cellular lysates, or lung tissue lysates obtained from different groups were subjected to SDS polyacrylamide gel electrophoresis (SDS-PAGE) and immunoblotting. The immunoreactive proteins were visualized with a chemiluminescent detection system (PerkinElmer Life Science, Inc. MA, USA) and BioMax LightFilm (Eastman Kodak Co., New Haven, CT, USA) according to the manufacturer's instructions.

### Measurement of mitochondria membrane potential

Previous report indicated that changes in mitochondria membrane potential during apoptosis affect the inner membrane and result in a loss of membrane potential. Membrane potential can be detected by incubation of cells with DiOC_6_. The interaction of mitochondria with DiOC6 is dependent on the stability of transmembrane potential and its uptake is reduced in apoptotic cells [28]. Therefore, apoptotic cells, with decreased mitochondrial membrane potential, showed reduced DiOC_6_ fluorescence. Changes in mitochondria membrane potential can be detected by flow cytometry. The treated Beas2B cells were washed, harvested, and re-suspended in PBS containing DiOC_6_ (final concentration is 40 nM). Cells were incubated for 15 min and the fluorescence was measured using the FACScan and analyzed using the FlowJo software.

### Statistical analyses

The results are expressed as the mean ± the standard deviation (SD). The experimental data were analyzed using Student's *t* test. Differences were considered statistically significant when the *p* value was less than 0.05.

## Results

### The tumor-promoting effect of TCDD on NNK-induced lung adenoma formation

To explore the role of TCDD in potentiating lung tumorigenesis, the effect of TCDD on NNK-induced lung tumorigenesis was analyzed by comparing female A/J mice treated with NNK(L) (low-dose NNK) + TCDD to those treated with NNK alone. The tumor incidence and multiplicity are listed in Table 1. The results showed a 20% lung tumor incidence in mice treated with TCDD alone and in the control group, and the tumor multiplicity was 0.25±0.05 and 0.1±0.02, respectively. The NNK(L) group showed a 50% tumor incidence and a tumor multiplicity of 0.85±0.05 (Table 1). The female A/J mice that received NNK(H) (high-dose NNK) as a positive lung tumorigenesis control showed the highest tumor incidence (100%) and multiplicity (3.5±0.1) of all the groups. Interestingly, the combination treatment of NNK(L) and TCDD significantly increased tumor incidence (90%) and multiplicity (2.2±0.2) compared with the control, TCDD alone, and NNK(L) groups (Table 1). These results indicated that TCDD alone did not induce lung tumors, but TCDD potentiated the tumor- promoting effect of NNK.

**Table 1:** The prevalence and tumor multiplicity of lung tumor formation in female A/J mice.

Treatment	Mice numbers	Tumor bearing mice	Prevalence (%)	Tumor multiplicity
1. Control	5	1	20	0.1±0.02
2. NNL (L)	10	5	50	0.85±0.05* ^,^ †
3. NNK (H)	10	10	100	3.5±0.1* ^,^ † ^,‡^
4. TCDD	10	2	20	0.25±0.05
5. NNK(L)+TCDD	10	9	90	2.2±0.2* ^,^ † ^,‡^

*Significantly higher (*p*<0.05) compared to Control groups.

† Significantly higher (*p*<0.05) compared to TCDD groups.

‡ Significantly higher (*p*<0.05) compared to NNK(L) groups.

The incidence and multiplicity of lung tumor formation in female A/J mice treated with low-dose NNK [NNK(L)], high-dose NNK [NNK(H)], TCDD, and TCDD combined with NNK(L). Tumor incidence is provided as the number of animals with tumors divided by the total number of animals at risk (%). Tumor multiplicity is the average tumor number observed in tumor-bearing mice. The data for lung tumor multiplicity are given as the mean ± SEM. *significantly higher (*p*<0.05) than the control group; ^†^significantly higher (*p*<0.05) than the TCDD group; ^‡^significantly higher (*p*<0.05) than the NNK(L) group.

### Proliferative and anti-apoptotic effect of TCDD *in vivo*

**Figure 2:**
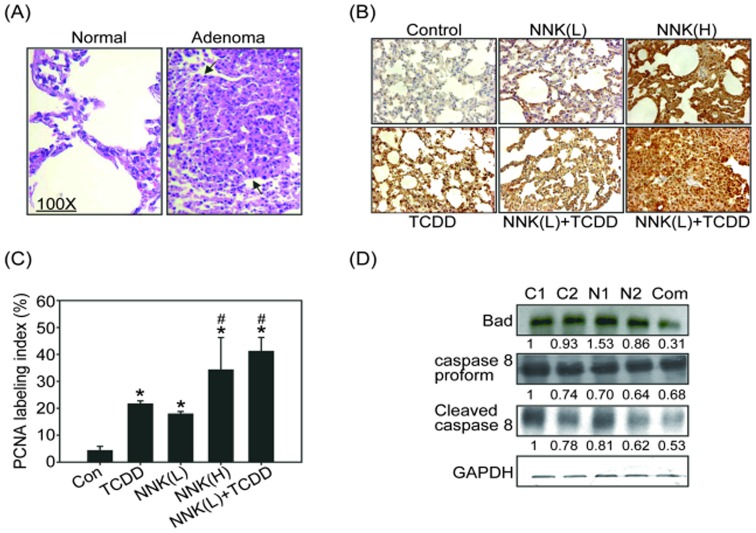
TCDD-induced cell proliferation in NNK pretreated mouse lung tissues. (A) The histological characteristics of mouse lungs stained with H&E from control and NNK treatment groups. Arrows indicate the adenocarcinoma in the lungs (magnification, X100). (B) Immunohistochemistry (IHC) was used to detect the expression of PCNA (cell proliferation marker) in mouse lung tissues. (C) Quantitative PCNA IHC data represent the mean ± SEM of three results in each group. *p<0.05, significantly higher than the control (Con) group; #*p*<0.05, significantly higher than the NNK(L) treatment group. (D) Analysis of pro-apoptotic proteins Bad and the precursor and cleaved form of caspase-8 in A/J mouse lung lysates treated with control (C1, C2), low-dose NNK (N1, N2), or NNK(L) combined with TCDD (Com). The results of three independent experiments using three individual mouse lungs were averaged. Representative data from one of the three independent experiments are shown. Equal protein loading was determined using a GAPDH antibody. The number below each line indicates the relative intensity of protein expression compared with that of the control group (defined as 1).

The histological features of normal lung tissue and lung adenomas induced by NNK are shown in Figure 2A. It has been indicated that NNK is a major contributor to lung carcinogenesis and significantly induced expression of PCNA (a marker of cell proliferation) in the lungs of A/J mice [29]. We proposed that TCDD would enhance cell proliferation thereby promoting lung tumor formation induced by NNK. PCNA expression was therefore analyzed using immunohistochemistry in the lung tissues of the A/J mice. As shown in Figures 2B and 2C, NNK and TCDD treatments slightly increased PCNA expression in lung tissues, whereas NNK(H) treatment induced approximately 40% PCNA positive staining in lung tissues, similar to the results observed in the group exposed to NNK(L) combined with TCDD. These results indicated that TCDD combined with low-dose NNK induced cell proliferation, and this may contribute, at least in part, to lung tumorigenic potency. In addition to cell proliferation enhanced by TCDD, apoptosis inhibition may also lead to tumorigenesis. We evaluated the expression of apoptosis regulators in lung tissue. Figure 2D shows that Bad and cleaved caspase-8 expression decreased in mice treated with low-dose NNK (N1, N2), whereas the TCDD combined with low-dose NNK treatment resulted in a pronounced inhibition of Bad and cleaved caspase-8 expression compared with the control (C1 and C2) and NNK(L)-treated groups (Figure 2D). These results suggested that one of the mechanisms of the TCDD-dependent promotion of NNK-induced lung tumorigenesis could be through the inhibition of apoptosis.

### TCDD attenuates staurosporine-induced apoptosis in Beas2B cells

**Figure 3:**
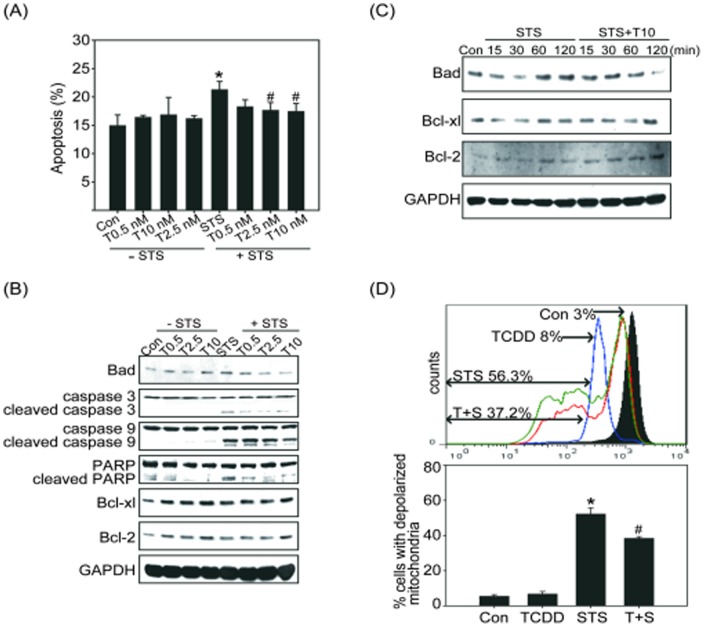
TCDD-attenuated STS-induced apoptosis in Beas2B cells. Beas2B cells were pretreated with TCDD (T, 0.5, 2.5, or 10 nM) for 4 h followed by 100 nM staurosporine (STS) for 2 h. (A) The percentage of apoptotic cells was determined by Annexin V staining and analyzed by flow cytometry. Mean ± SEM; n = 3; *p<0.05 compared with the control (Con) group; #*p*<0.05 compared with the STS treatment alone group. (B) Beas2B cells treated with 0.5, 2.5, or 10 nM TCDD for 4 h alone (left panel) or followed by 100 nM STS for 2 h (right panel). The expression of the apoptotic regulators Bad, PARP, cleaved caspase-3 and -9 and the anti-apoptotic proteins Bcl-xl and Bcl-2 were analyzed by western blotting analysis. (C) Beas2B cells were treated with STS alone (left panel) or pretreated with TCDD followed by STS treatment for the indicated time, and cell lysates were then isolated and immunoblotted with anti-Bad, Bcl-xl, and Bcl-2 antibodies. The membranes were probed with an anti-GAPDH antibody to confirm equal protein loading. Representative data from one of three independent experiments are shown. (D) Beas2B cells were treated with DMSO (control, Con), 10 nM TCDD, 100 nM STS, or pretreated with TCDD for 4 h followed by STS treatment for 24 h (T+S). Cells were incubated with DiOC_6_ for 15 min, and the loss of mitochondria membrane potential levels were detected by flow cytometry. The upper panel indicated fluorescence histogram of Beas2B cells stained with DiOC_6_. The filled tracing indicated the control groups. The open tracing with blue, green, and red lines indicated the TCDD groups, STS groups, and TCDD+STS groups, respectively. Histograms depicted all the acquired events. The lower panel indicated the quantification of cells with depolarized mitochondria. Numbers refer to the percentage of apoptotic cells in each sample. Mean ± SEM; n = 3; *p<0.05 compared with the Con group; #*p*<0.05 compared with the STS treatment alone group.

To further address the effect of TCDD on apoptosis inhibition, we performed a series of mechanistic experiments *in vitro*. TCDD has been reported to inhibit STS-induced apoptosis in normal mammary gland epithelial cells [30]. Therefore, we examined the anti-apoptotic effect of TCDD in normal human bronchial epithelial cells treated with the apoptosis inducer STS. The level of apoptosis was determined by an Annexin V binding assay. The results showed that the basal rate of apoptosis in control cells was approximately 13% and that STS induced apoptosis in approximately 22% of Beas2B cells (Figure 3A). Pretreatment with TCDD (10 nM) resulted in a significant suppression of STS-induced apoptosis: the combined treatment only caused approximately 17.2% apoptosis in the Beas2B cells (Figure 3A). The downstream mediators of apoptosis, such as cleaved caspase-3, caspase-9, and PARP, also decreased in response to TCDD pretreatment followed by STS treatment (Figure 3B, right panel). The inhibition of apoptosis was associated with a dose dependent the up-regulation of the anti-apoptotic proteins Bcl-xl and Bcl-2 after TCDD treatment (Figure 3B, left panel). These effects were also observed in the cultures treated with both TCDD (10 nM) and STS (Figure 3B, right panel). Moreover, pretreatment with TCDD resulted in a time-dependent down-regulation of Bad and up-regulation of Bcl-xl and Bcl-2 after STS treatment (Figure 3C). Previous studies indicated loss of mitochondria membrane integrity is one of the early events leading to apoptosis [28]. To evaluate the effects of TCDD on preventing mitochondria membrane potential loss in response to STS, cells were pretreated with the fluorescent mitochondria staining dye DiOC_6_, and the changes of mitochondrial membrane potential were measured by flow cytometry. As shown in Figure 3D, a marked increases of cells with depolarized mitochondria revealed by staining with DiOC_6_ was occurred in Beas2B cells treated with STS for 24 h (56.3%). The increases of depolarized mitochondria were reduced by pretreatment with TCDD 10 nM for 4 h followed by STS treatment for 24 h (37.2%). These results demonstrated that TCDD exerts an anti-apoptotic effect in lung epithelial cells in response to a DNA-damaging agent.

### TCDD-mediated inhibition of apoptosis depends on Akt and ERK1/2 activation

**Figure 4:**
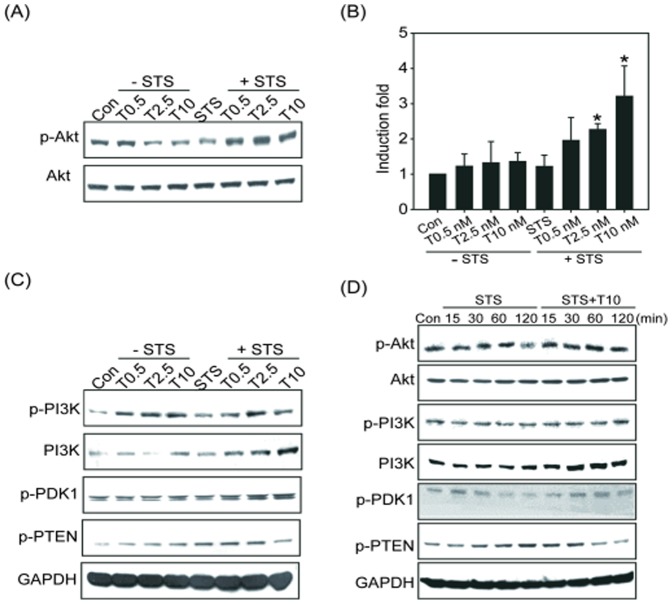
Effect of STS, TCDD or the combination on the expression of AKT and regulatory factors. (A) Beas2B cells were treated with TCDD (0.5, 2.5, or 10 nM) alone or in combination with STS, and the activation of Akt was determined by detecting phosphorylated Akt. The membranes were probed with an anti-Akt antibody to confirm equal protein loading. (B) The fold induction of phosphorylated Akt was quantitated and is represented by the mean ± SEM of three results in each group. *p<0.05, significantly higher than STS group. The fold change was corrected for the amount of Akt loaded. (C) The protein levels of p-PI3K, PI3K, p-PDK1, and p-PTEN were analyzed by western blot following treatment with TCDD alone or in combination with STS. (D) Beas2B cells were treated with STS alone or TCDD in combination with STS for the indicated time, and the cell lysates were then isolated and immunoblotted with anti-p-Akt, Akt, p-PI3K, PI3K, p-PDK1, and p-PTEN antibodies. The membranes were probed with an anti-GAPDH antibody to confirm equal protein loading. Representative data from one of three independent experiments are shown.

We next analyzed the underlying molecular mechanism of the TCDD-regulated inhibition of apoptosis. It is known that the PI3K/Akt pathway mediates both cell survival and the inhibition of apoptosis [31]. We hypothesized that TCDD would induce the phosphorylation in the PI3K/Akt pathway and consequently lead to the inhibition of apoptosis. The level of phosphorylated Akt was therefore assessed. We found a decrease in Akt phosphorylation in the Beas2B cells treated with 100 nM STS for 24 h compared with the control group. In the presence of TCDD, the phosphorylation of Akt was significantly increased compared with the control and STS-treated groups (Figure 4A and 4B). The upstream regulators of Akt were also analyzed, and the results showed that STS inhibited the phosphorylation of PDK1 at the early time points (120 min, Figure 4D), whereas it increased the phosphorylation of PTEN, a negative regulator of Akt, between 60 min and 24 h (Figure 4C and 4D). In contrast, pretreatment with TCDD significantly activated PI3K and PDK1 and inhibited PTEN in a dose-dependent manner (Figure 4C). A time-course study indicated that pretreatment with TCDD followed by STS treatment induced the activation of Akt and PI3K at 30 min and inhibited the activation of PTEN at 60 and 120 min (Figure 4D). These results suggested that TCDD might increase Akt activation by regulating the upstream regulators of Akt, specifically by activating PI3K and PDK1 and the down-regulating PTEN.

**Figure 5:**
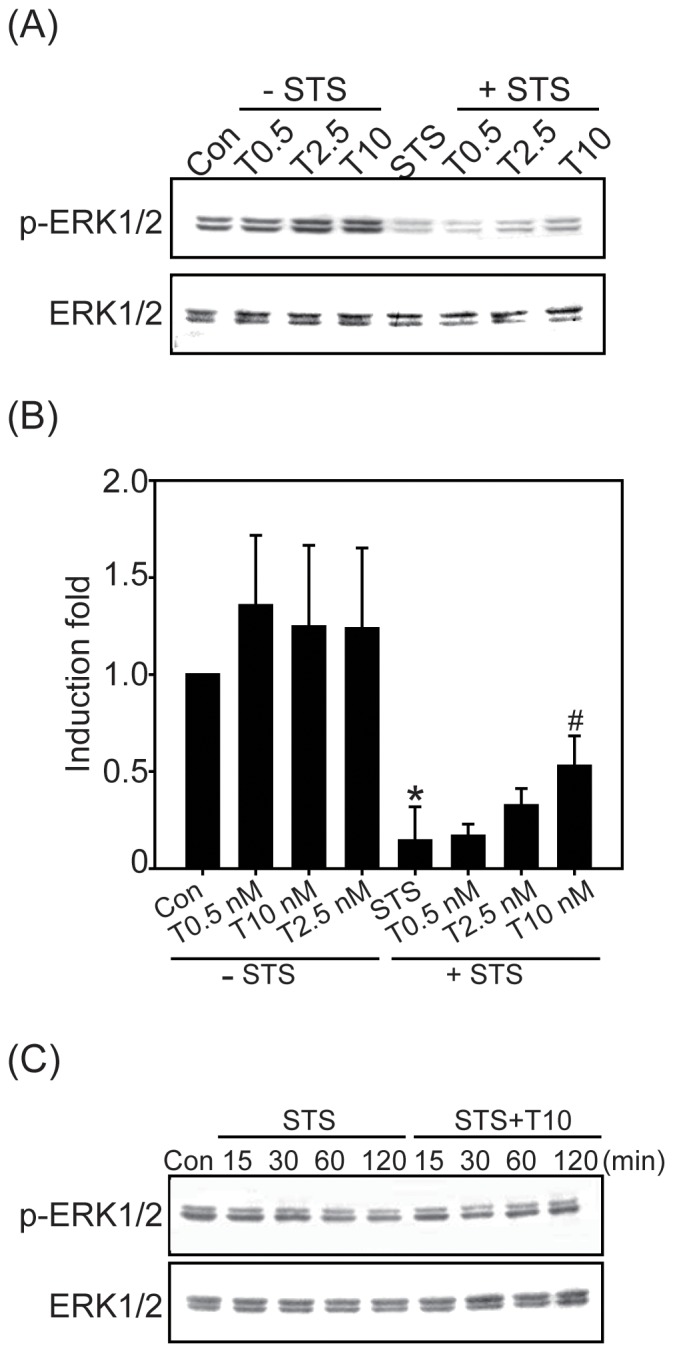
Effect of STS, TCDD or the combination on ERK1/2 activation. (A) Beas2B cells were treated with TCDD (0.5, 2.5, or 10 nM) alone or in combination with 100 nM STS for 2 h, and p-ERK1/2 was determined by western blotting analysis. (B) The fold induction of ERK1/2 activity was quantified and is represented by the mean ± SEM of three results in each group. *p<0.05, significantly lower than the control (Con) group; #*p*<0.05 compared with the STS treatment alone group. (C) Beas2B cells were treated with STS alone or TCDD in combination with STS for 15, 30, 60, or 120 min, and cell lysates were then isolated and immunoblotted with the anti-p-ERK1/2 antibody. The membranes were probed with an anti-ERK1/2 antibody to confirm equal proteins loading. Representative data from one of three independent experiments are shown.

In addition to Akt, ERK1/2 has also been shown to inhibit apoptosis [32]. We therefore analyzed the activation of the ERK1/2 signaling pathway. As shown in Figure 5A and 5B, the expression of phosphorylated ERK1/2 was significantly down-regulated in Beas2B cells treated with STS, whereas TCDD treatment alone increased the activation of ERK1/2 (Figure 5A and 5B). Pretreatment with TCDD prevented the STS-inhibited ERK1/2 activation in a dose- and time-dependent manner (Figure 5A and 5C). These results indicated that ERK1/2 might play an important role in the TCDD-mediated inhibition of STS-induced apoptosis.

**Figure 6:**
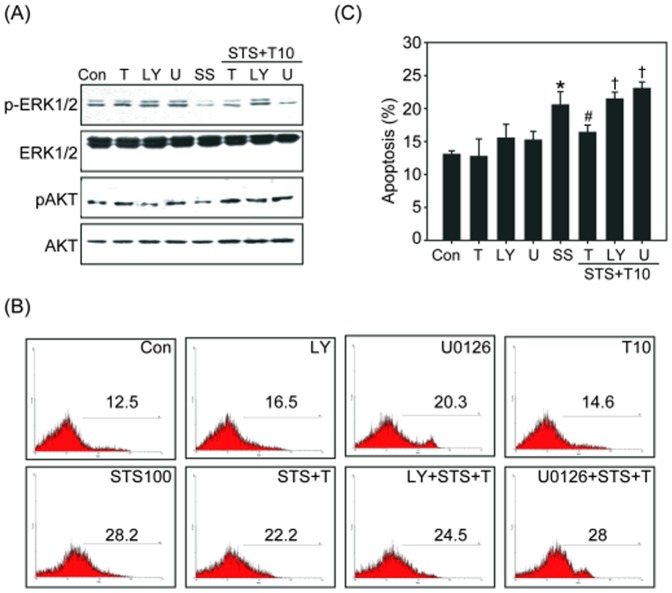
Effect of LY294002(LY) or U0126(U) on the anti-apoptotic activity of TCDD. Beas2B cells were pretreated with 10 µM LY or 100 nM U for 1 h, then treated with TCDD (10 nM) for 4 h, followed by 100 nM STS for 2 h. (A) The expression of p-ERK1/2, ERK1/2, p-Akt, and Akt in different treatment groups was determined by western blotting analysis. Representative data from one of three independent experiments are shown. (B) The level of apoptosis after treatment with LY or U was measured by Annexin V staining and analyzed by flow cytometry, and the quantified results are shown (C). The data represent the mean ± SEM of three independent experiments; *p<0.05 compared with the control (Con) group; #*p*<0.05 compared with the STS-treated group, †*p*<0.05 compared with the TCDD + STS group.

Chemical inhibitors were also utilized to further confirm the requirement of Akt and ERK1/2 activity in the TCDD-dependent inhibition of apoptosis. Beas2B cells were pretreated with the PI3K/Akt inhibitor Ly294002 (Ly) or with the MAPK/ERK1/2 inhibitor U0126 (U) for 1 h, followed by treatment with TCDD and STS. As shown in Figure 6A, these chemical inhibitors reduced the expression of phosphorylated Akt or ERK1/2, respectively. Treatment with LY or U0126 alone slightly increased apoptosis in Beas2B cells. Pretreatment with LY or U0126 blocked the anti-apoptotic effect of TCDD on STS-induced apoptosis (Figure 6B and 6C). The level of apoptosis in cells pretreated with LY or U0126 prior to treatment with TCDD and STS increased apoptosis to 25% or 28%, respectively (Figure 6C). These results revealed that both the PI3K/Akt and ERK1/2 signaling pathways are involved in the TCDD-mediated inhibition of STS-induced apoptosis.

## Discussion

TCDD has been designated a human carcinogen by the IARC based on animal and mechanistic studies. TCDD exposure significantly increases the risk of lung cancer, non-Hodgkin's lymphoma, soft tissue sarcoma, and many other cancer types [6], [8]. Although the role of TCDD in carcinogenesis is well accepted, the molecular mechanism of TCDD-induced carcinogenicity remains unclear. TCDD is regarded as the most potent liver tumor promoter in rodent models [2]. Consistent with this concept, our previous report demonstrated a potential mechanism by which TCDD may act as a lung tumor promoter in an *in vivo* animal model [15]. In addition, study that used DEN as an initiator and TCDD as a promoter also showed that continuous TCDD exposure is required to promote lung tumorigenesis [1]. Thus, the tumor-promoting effect of TCDD could be the cause of its carcinogenicity, suggesting that TCDD acts as a co-carcinogen. In the present study, we further demonstrated that TCDD combined with low-dose NNK induced lung tumors in A/J mice to the same extent as high-dose NNK (Table 1). We also found that TCDD can potentiate the cell proliferation induced by NNK as determined by PCNA staining (Figure 2). These results are consistent with previous reports that TCDD increased cell cycle progression in keratinocytes and hepatocytes [33]. Additional mechanistic studies indicated that AhR signaling activated by TCDD induces the expression of DP2 (DNA polymerase large subunit) and PCNA, thereby enhanced the activity of E2F leading to increased DNA synthesis in A549 cells [34]. Consequently, the increased cell proliferation triggered by TCDD may contribute to its lung tumor promotion effect when combined with NNK.

It is believed that certain hallmark capabilities are shared by all tumors. These hallmarks include insensitivity to antigrowth signals, which leads to continuous cell cycle progression, self-sufficiency in growth signals, unlimited replicative potential, and resistance to apoptosis [35]. Previous reports have shown the anti-apoptotic effect of TCDD after treatment with a variety of cell-damaging agents, including UV, H_2_O_2_, STS, γ-irradiation, TGF-β, and paraquat [30]. TCDD is reported to inhibit apoptosis in liver and mammary cells, which may explain the increased risk for liver and breast cancer following TCDD exposure in humans [19]. The inhibition of spontaneous and chemically induced apoptosis in rodent livers is the underlying mechanism for the carcinogenicity of TCDD [2]. TCDD also inhibits EGF withdrawal-induced apoptosis in the normal human mammary epithelial cell line MCF-10A [17]. These studies have provided clues regarding the mechanism of the TCDD-dependent inhibition of apoptosis in damaged cells and the subsequent promotion of tumor growth. Therefore, in addition to the increased cell cycle progression induced by TCDD, we propose that the inhibition of lung epithelial cell apoptosis could be one of the causative events in lung tumor promotion by TCDD. This inhibition could allow genetically aberrant cells to evade apoptosis. Although multiple pathways have been suggested to play salient roles in apoptosis inhibition by TCDD, the exact anti-apoptotic mechanism of TCDD remains unclear. Thus, we designed a series of experiments in which human normal lung epithelial cells were treated with the apoptosis inducer STS and/or TCDD, to identify the signaling pathways involved in the TCDD-regulated inhibition of apoptosis. Consistent with the previous data, our current findings revealed that STS-induced apoptosis in human normal bronchial epithelial Beas2B cells could be attenuated by pretreatment with TCDD. The mechanistic studies indicated that the STS-induced activation of Bad, caspase-9, and caspase-3 were down-regulated by treatment with TCDD (Figure 3), which supports the hypothesis that TCDD can inhibit apoptosis.

Using pathway-specific chemical inhibitors, we identified the possible molecular signaling pathways that are involved in the TCDD-mediated inhibition of apoptosis: the Akt and ERK1/2 signaling pathways (Figure 4–6). Consistent with our data, the c-Src kinase inhibitors PP-2 and CGP77675 and the ERK1/2 inhibitors PD98059 and U0126 reversed the inhibition of apoptosis by TCDD in the human mammary epithelial cell line MCF-10A, whereas the p38 inhibitor SB202190 and the JNK inhibitor SP600125 did not [30]. TCDD is also known to induce PKC and ERK1/2 activation by an AhR-independent mechanism [36], [37]. In addition, Pierre et al. indicated that TCDD treatment leads to an activated Ras-GTP state, ERK1/2 activation, and accelerated cell proliferation in human hepatoma HepG2 cell lines [38]. In human mammary gland epithelial cells, TCDD antagonized apoptosis by mimicking the action of EGF through the activation of the c-Src/ERK1/2 signaling pathway [30]. Consistent with these results, our current finding indicated that TCDD can trigger its anti-apoptotic effect through ERK1/2-mediated pathways in Beas2B cells.

In addition to ERK1/2 activation, we also observed that Akt and its upstream mediators were also affected by TCDD. It has recently been reported that the major actions of the PI3K/Akt signaling pathway are to evoke cell proliferation and inhibit apoptosis or autophagic cell death [39]. These signaling pathways act as a cellular defense mechanism that rescues damaged cells from apoptosis thereby promoting tumorigenesis. The results in Figure 4 show that DNA damaging agent STS significantly inhibited Akt and led to apoptosis induction in Beas2B cells, whereas pretreatment with TCDD attenuated the apoptosis induction (Figure 4). Regarding the possible role of PI3K/Akt in apoptosis inhibition, PI3K/Akt might antagonize apoptosis by phosphorylating the anti-apoptotic protein Bcl-2 and pro-apoptotic protein Bad through activation of NFκB [19]. In addition, downstream substrates, including Bim, caspase-9, FOXO3a, MDM-2, p21, and p27, are regulated by Akt, indicating that Akt is a critical growth regulatory switch [40]. In the present study, we also found that the upstream mediators of Akt such as PI3K and PDK were activated in response to TCDD, whereas the Akt phosphatase PTEN was down-regulated when the cells were treated with TCDD combined with STS (Figure 4). PTEN is an important negative regulator of the PI3K pathway. PTEN can regulate cell division and also direct cells to enter a cell death pathway when sufficient growth has taken place by inducing cell cycle arrest through the Rb protein. Therefore, PTEN inactivation leads to over-activation of Akt, which in turn, leads to uncontrolled cell growth, decreased apoptosis, and enhanced tumorigenesis [41]. Thus, we suggest that one of the new findings in the current study is that the anti-apoptotic action of TCDD is also mediated by the upstream component of Akt such as regulation of PDK1 and PTEN. This finding is important in the context of the observations that PTEN loss or inactivation is associated with various cancers in lung, breast, kidney, and prostate [41]. We suggest that TCDD exposure-induced Akt activation may be a consequence of PDK1 activation or PTEN inactivation. Indeed, PDK1 is a transducer of PI3K signaling pathway and is a critical component of oncogenic PI3K/Akt activity in some cancers [42]. Class I PI3K is activated by cell surface receptors such as epidermal growth factor receptor (EGFR), platelet-derived growth factor receptor (PDGFR), vascular endothelial growth factor receptor (VEGFR), insulin-like growth factor (IGF) and so on [43]. Interestingly, previous reports indicated TCDD stimulated EGFR activation in cancer cells [44], and TCDD-activated AhR can cross talk with EGFR signaling [45]. These reports indicated that TCDD can activate PI3K signaling through receptors directly or through AhR-dependent signaling pathways, therefore, the role of TCDD in lung tumorigenesis by regulation of PI3K/Akt upstream receptors is worthy of further studies in the future.

Recent surveys found high levels of polychlorinated biphenyl (PCBs) contamination in sea food, milk, eggs, herbicides, and sediment [3]. Among these dioxin-like compounds, TCDD is thought to be the most toxic congener based on a variety of animal studies. These reports implied that human may be persistently exposed to TCDD in daily life through food consumption or environmental contamination. As a result, the accumulation of TCDD increases the possibility for synergistic interactions with other hazardous chemicals, potentially leading to severe toxicity including tumorigenesis. Understanding the tumorigenic mechanisms by TCDD may be useful to provide a validate cancer preventive target for chemopreventive agents. In the present study, we have shown that TCDD induced cell proliferation and promoted NNK-induced lung tumor formation in A/J mice. Moreover, TCDD rescued Beas2B cells from apoptosis induced by STS. The mechanism of inhibition of apoptosis is likely mediated through the activation of the PI3K/Akt and ERK1/2 pathways, as demonstrated by the effectiveness of pathway-specific inhibitors in abrogating the anti-apoptotic effect of TCDD (Figure 4-6). These observations support the hypothesis that the ability of TCDD to promote lung tumorigenesis is largely dependent on both cell proliferation and apoptosis inhibition. The current results imply that there is a close relationship among PI3K/PDK/Akt activation, ERK1/2 activation, PTEN inactivation, and apoptosis inhibition triggered by TCDD. PI3K/Akt is a major mediator of cell proliferation and plays important roles in tumorigenesis. Due to the fact that most of the biological effects of TCDD require the activation of AhR, which results in transcriptional activation or the repression of diverse array of genes [7]. Thus, there is a need in the future to determine whether TCDD-induced PI3K/Akt, PDK1, and ERK1/2 activation depends on AhR-mediated signaling. In addition, AhR has been reported that possesses a promiscuous ligand binding site, which could be targeted by numerous dietary bioactive compounds (found in grapefruit juice, potatoes, and cruciferous vegetables), leading to the interference of gene expression targeted by the AhR [46]. Whether those bioactive food components could modulate the TCDD-activated AhR and the consequent lung tumorigenesis deserves further investigation.
